# Supplementary material for: An open-source framework for large-scale, flexible evaluation of biomedical text mining systems
Source: J Biomed Discov Collab. 2008 Jan 29;3:1. doi: 10.1186/1747-5333-3-1 (PMC2276192; doi:10.1186/1747-5333-3-1)
Supplement: Additional file 1 — Evaluation platform source code. The additional file contains the software evaluation framework discussed in this paper. The most current release can be downloaded from SourceForge.net [1]. See the accompanying README file for instructions on installation and use. Also included in the distribution is a collection of UIMA wrappers for some commonly used BioNLP tools and annotated corpora. [file 1747-5333-3-1-S1.zip › bionlp_uima_repository-1.0.1/docs/api/src-html/edu/uchsc/ccp/uima/mention/CCPClassMention_Type.html]

```
001    
002    /* First created by JCasGen Tue Aug 28 16:44:10 MDT 2007 */
003    package edu.uchsc.ccp.uima.mention;
004    
005    import org.apache.uima.cas.Feature;
006    import org.apache.uima.cas.FeatureStructure;
007    import org.apache.uima.cas.Type;
008    import org.apache.uima.cas.impl.CASImpl;
009    import org.apache.uima.cas.impl.FSGenerator;
010    import org.apache.uima.cas.impl.FeatureImpl;
011    import org.apache.uima.cas.impl.TypeImpl;
012    import org.apache.uima.jcas.JCas;
013    import org.apache.uima.jcas.JCasRegistry;
014    
015    /** The CCP ClassMention is the root of a flexible class structure that can be used to store virtually any frame-based representation of a particular class. Common class mention types include, but are not limited to, such things as entities (protein, cell type, cell line, disease, tissue, etc.) and frames (interaction, transport, regulation, etc.).
016     * Updated by JCasGen Tue Aug 28 16:44:10 MDT 2007
017     * @generated */
018    public class CCPClassMention_Type extends CCPMention_Type {
019      /** @generated */
020      protected FSGenerator getFSGenerator() {return fsGenerator;}
021      /** @generated */
022      private final FSGenerator fsGenerator = 
023        new FSGenerator() {
024          public FeatureStructure createFS(int addr, CASImpl cas) {
025                             if (CCPClassMention_Type.this.useExistingInstance) {
026                               // Return eq fs instance if already created
027                         FeatureStructure fs = CCPClassMention_Type.this.jcas.getJfsFromCaddr(addr);
028                         if (null == fs) {
029                           fs = new CCPClassMention(addr, CCPClassMention_Type.this);
030                               CCPClassMention_Type.this.jcas.putJfsFromCaddr(addr, fs);
031                               return fs;
032                         }
033                         return fs;
034            } else return new CCPClassMention(addr, CCPClassMention_Type.this);
035              }
036        };
037      /** @generated */
038      public final static int typeIndexID = CCPClassMention.typeIndexID;
039      /** @generated 
040         @modifiable */
041      public final static boolean featOkTst = JCasRegistry.getFeatOkTst("edu.uchsc.ccp.uima.mention.CCPClassMention");
042     
043      /** @generated */
044      final Feature casFeat_slotMentions;
045      /** @generated */
046      final int     casFeatCode_slotMentions;
047      /** @generated */ 
048      public int getSlotMentions(int addr) {
049            if (featOkTst && casFeat_slotMentions == null)
050          jcas.throwFeatMissing("slotMentions", "edu.uchsc.ccp.uima.mention.CCPClassMention");
051        return ll_cas.ll_getRefValue(addr, casFeatCode_slotMentions);
052      }
053      /** @generated */    
054      public void setSlotMentions(int addr, int v) {
055            if (featOkTst && casFeat_slotMentions == null)
056          jcas.throwFeatMissing("slotMentions", "edu.uchsc.ccp.uima.mention.CCPClassMention");
057        ll_cas.ll_setRefValue(addr, casFeatCode_slotMentions, v);}
058        
059       /** @generated */
060      public int getSlotMentions(int addr, int i) {
061            if (featOkTst && casFeat_slotMentions == null)
062          jcas.throwFeatMissing("slotMentions", "edu.uchsc.ccp.uima.mention.CCPClassMention");
063        if (lowLevelTypeChecks)
064          return ll_cas.ll_getRefArrayValue(ll_cas.ll_getRefValue(addr, casFeatCode_slotMentions), i, true);
065        jcas.checkArrayBounds(ll_cas.ll_getRefValue(addr, casFeatCode_slotMentions), i);
066            return ll_cas.ll_getRefArrayValue(ll_cas.ll_getRefValue(addr, casFeatCode_slotMentions), i);
067      }
068       
069      /** @generated */ 
070      public void setSlotMentions(int addr, int i, int v) {
071            if (featOkTst && casFeat_slotMentions == null)
072          jcas.throwFeatMissing("slotMentions", "edu.uchsc.ccp.uima.mention.CCPClassMention");
073        if (lowLevelTypeChecks)
074          ll_cas.ll_setRefArrayValue(ll_cas.ll_getRefValue(addr, casFeatCode_slotMentions), i, v, true);
075        jcas.checkArrayBounds(ll_cas.ll_getRefValue(addr, casFeatCode_slotMentions), i);
076        ll_cas.ll_setRefArrayValue(ll_cas.ll_getRefValue(addr, casFeatCode_slotMentions), i, v);
077      }
078     
079     
080      /** @generated */
081      final Feature casFeat_ccpTextAnnotations;
082      /** @generated */
083      final int     casFeatCode_ccpTextAnnotations;
084      /** @generated */ 
085      public int getCcpTextAnnotations(int addr) {
086            if (featOkTst && casFeat_ccpTextAnnotations == null)
087          jcas.throwFeatMissing("ccpTextAnnotations", "edu.uchsc.ccp.uima.mention.CCPClassMention");
088        return ll_cas.ll_getRefValue(addr, casFeatCode_ccpTextAnnotations);
089      }
090      /** @generated */    
091      public void setCcpTextAnnotations(int addr, int v) {
092            if (featOkTst && casFeat_ccpTextAnnotations == null)
093          jcas.throwFeatMissing("ccpTextAnnotations", "edu.uchsc.ccp.uima.mention.CCPClassMention");
094        ll_cas.ll_setRefValue(addr, casFeatCode_ccpTextAnnotations, v);}
095        
096       /** @generated */
097      public int getCcpTextAnnotations(int addr, int i) {
098            if (featOkTst && casFeat_ccpTextAnnotations == null)
099          jcas.throwFeatMissing("ccpTextAnnotations", "edu.uchsc.ccp.uima.mention.CCPClassMention");
100        if (lowLevelTypeChecks)
101          return ll_cas.ll_getRefArrayValue(ll_cas.ll_getRefValue(addr, casFeatCode_ccpTextAnnotations), i, true);
102        jcas.checkArrayBounds(ll_cas.ll_getRefValue(addr, casFeatCode_ccpTextAnnotations), i);
103            return ll_cas.ll_getRefArrayValue(ll_cas.ll_getRefValue(addr, casFeatCode_ccpTextAnnotations), i);
104      }
105       
106      /** @generated */ 
107      public void setCcpTextAnnotations(int addr, int i, int v) {
108            if (featOkTst && casFeat_ccpTextAnnotations == null)
109          jcas.throwFeatMissing("ccpTextAnnotations", "edu.uchsc.ccp.uima.mention.CCPClassMention");
110        if (lowLevelTypeChecks)
111          ll_cas.ll_setRefArrayValue(ll_cas.ll_getRefValue(addr, casFeatCode_ccpTextAnnotations), i, v, true);
112        jcas.checkArrayBounds(ll_cas.ll_getRefValue(addr, casFeatCode_ccpTextAnnotations), i);
113        ll_cas.ll_setRefArrayValue(ll_cas.ll_getRefValue(addr, casFeatCode_ccpTextAnnotations), i, v);
114      }
115     
116    
117    
118    
119      /** initialize variables to correspond with Cas Type and Features
120            * @generated */
121      public CCPClassMention_Type(JCas jcas, Type casType) {
122        super(jcas, casType);
123        casImpl.getFSClassRegistry().addGeneratorForType((TypeImpl)this.casType, getFSGenerator());
124    
125     
126        casFeat_slotMentions = jcas.getRequiredFeatureDE(casType, "slotMentions", "uima.cas.FSArray", featOkTst);
127        casFeatCode_slotMentions  = (null == casFeat_slotMentions) ? JCas.INVALID_FEATURE_CODE : ((FeatureImpl)casFeat_slotMentions).getCode();
128    
129     
130        casFeat_ccpTextAnnotations = jcas.getRequiredFeatureDE(casType, "ccpTextAnnotations", "uima.cas.FSArray", featOkTst);
131        casFeatCode_ccpTextAnnotations  = (null == casFeat_ccpTextAnnotations) ? JCas.INVALID_FEATURE_CODE : ((FeatureImpl)casFeat_ccpTextAnnotations).getCode();
132    
133      }
134    }
135    
136    
137    
138
```
